# Breast cancer risk and prevention in 2024: An overview from the Breast Cancer UK ‐ Breast Cancer Prevention Conference

**DOI:** 10.1002/cam4.70255

**Published:** 2024-09-24

**Authors:** Britta Stordal, Michelle Harvie, Michael N. Antoniou, Michelle Bellingham, Doris S. M. Chan, Philippa Darbre, Oskar Karlsson, Andreas Kortenkamp, Pamela Magee, Stefano Mandriota, Elisabete Silva, James E. Turner, Laura N. Vandenberg, D. Gareth Evans

**Affiliations:** ^1^ Department of Natural Sciences Middlesex University London, The Burroughs Hendon London UK; ^2^ Manchester University Hospital Foundation NHS Trust Manchester UK; ^3^ Division of Cancer Sciences, Faculty of Biology, Medicine and Health University of Manchester Manchester UK; ^4^ Gene Expression and Therapy Group, Faculty of Life Sciences & Medicine, Department of Medical and Molecular Genetics King's College London London UK; ^5^ School of Biodiversity, One Health and Veterinary Medicine College of Medical, Veterinary and Life Sciences, University of Glasgow Glasgow UK; ^6^ Department of Epidemiology and Biostatistics, School of Public Health Imperial College London London UK; ^7^ School of Biological Sciences University of Reading Reading UK; ^8^ Science for Life Laboratory, Department of Environmental Science Stockholm University Stockholm Sweden; ^9^ Centre for Pollution Research and Policy, Department of Life Sciences College of Health, Medicine and Life Sciences, Brunel University London Uxbridge UK; ^10^ Nutrition Innovation Centre for Food & Health (NICHE) Ulster University Coleraine UK; ^11^ Laboratoire de Cancérogenèse Environnementale, Fondation des Grangettes Chêne‐Bougeries Switzerland; ^12^ The Francis Crick Institute London UK; ^13^ School of Sport, Exercise and Rehabilitation Sciences University of Birmingham Birmingham UK; ^14^ Department of Environmental Health Sciences, School of Public Health and Health Sciences University of Massachusetts Amherst Amherst Massachusetts USA; ^15^ Division of Evolution, Infection and Genomics, School of Biological Sciences, Faculty of Biology, Medicine and Health University of Manchester Manchester UK

**Keywords:** aluminium, breast cancer, breastfeeding, endocrine disrupting chemicals, epidemiology, exercise, health behaviour, pregnancy exposure, prevention, risk, weight

## Abstract

The Breast Cancer UK—Breast Cancer Prevention Conference addressed risk from environmental pollutants and health behaviour‐related breast‐cancer risk. Epidemiological studies examining individual chemicals and breast cancer risk have produced inconclusive results including endocrine disrupting chemicals (EDCs) Bisphenol A, per‐ and polyfluorinated alkyl substances as well as aluminium. However, laboratory studies have shown that multiple EDCs, can work together to exhibit effects, even when combined at levels that alone are ineffective. The TEXB‐α/β assay measures total estrogenic load, and studies have provided evidence of a link between multiple‐chemical exposures and breast cancer. However, prospective studies using TEXB‐α/β are needed to establish a causative link. There is also a need to assess real‐life exposure to environmental‐chemical mixtures during pregnancy, and their potential involvement in programming adverse foetal health outcomes in later life.

Higher rates of breast cancer have occurred alongside increases in potentially‐modifiable risk factors such as obesity. Increasing body‐mass index is associated with increased risk of developing postmenopausal breast cancer, but with decreased risk of premenopausal breast cancer. In contrast, lower rates of breast cancer in Asian compared to Western populations have been linked to soya/isoflavone consumption. Risk is decreased by breastfeeding, which is in addition to the decrease in risk observed for each birth and a young first‐birth. Risk is lower in those with higher levels of self‐reported physical activity. Current evidence suggests breast‐cancer survivors should also avoid weight gain, be physically active, and eat a healthy diet for overall health.

A broad scientific perspective on breast cancer risk requires focus on both environmental exposure to chemicals and health behaviour‐related risk. Research into chemical exposure needs to focus on chemical mixtures and prospective epidemiological studies in order to test the effects on breast cancer risk. Behaviour‐related research needs to focus on implementation as well as deeper understanding of the mechanisms of cancer prevention.

## Graphical abstract

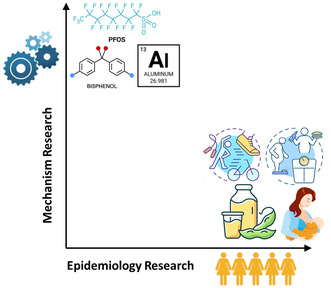

## 1 BACKGROUND

Worldwide there were over 2.3 million new cases and 685,000 deaths from breast cancer in 2020.^1^ By 2040, breast cancer incidence is projected to grow by over 40%, to about 3 million cases every year due to population growth and ageing. Similarly, deaths from breast cancer will increase by over 50%, to about 1 million in 2040.^1^ Current primary prevention strategies are focused on health behaviour‐related factors such as asking women to reduce their weight, exercise more and drink less alcohol. While there is very good evidence that adult weight gain,^2^, ^3^ lack of physical activity^4^, ^5^ and alcohol consumption are breast cancer risk factors, this advice to women has essentially remained unchanged for many decades. Unfortunately, health behaviour‐related advice has had a limited impact on the rise of breast cancer cases due to challenges with implementation in the general population.

The recent Breast Cancer UK—Breast Cancer Prevention Conference hosted at Middlesex University London discussed breast cancer prevention research based on a wide perspective. While maintaining the focus on health behaviour‐related breast cancer risk, the conference also included environmental pollutants that reach women via their diet, inhalation or dermal uptake. In particular, it focused on chemical exposures that occur during foetal development and puberty.

**FIGURE 1:**
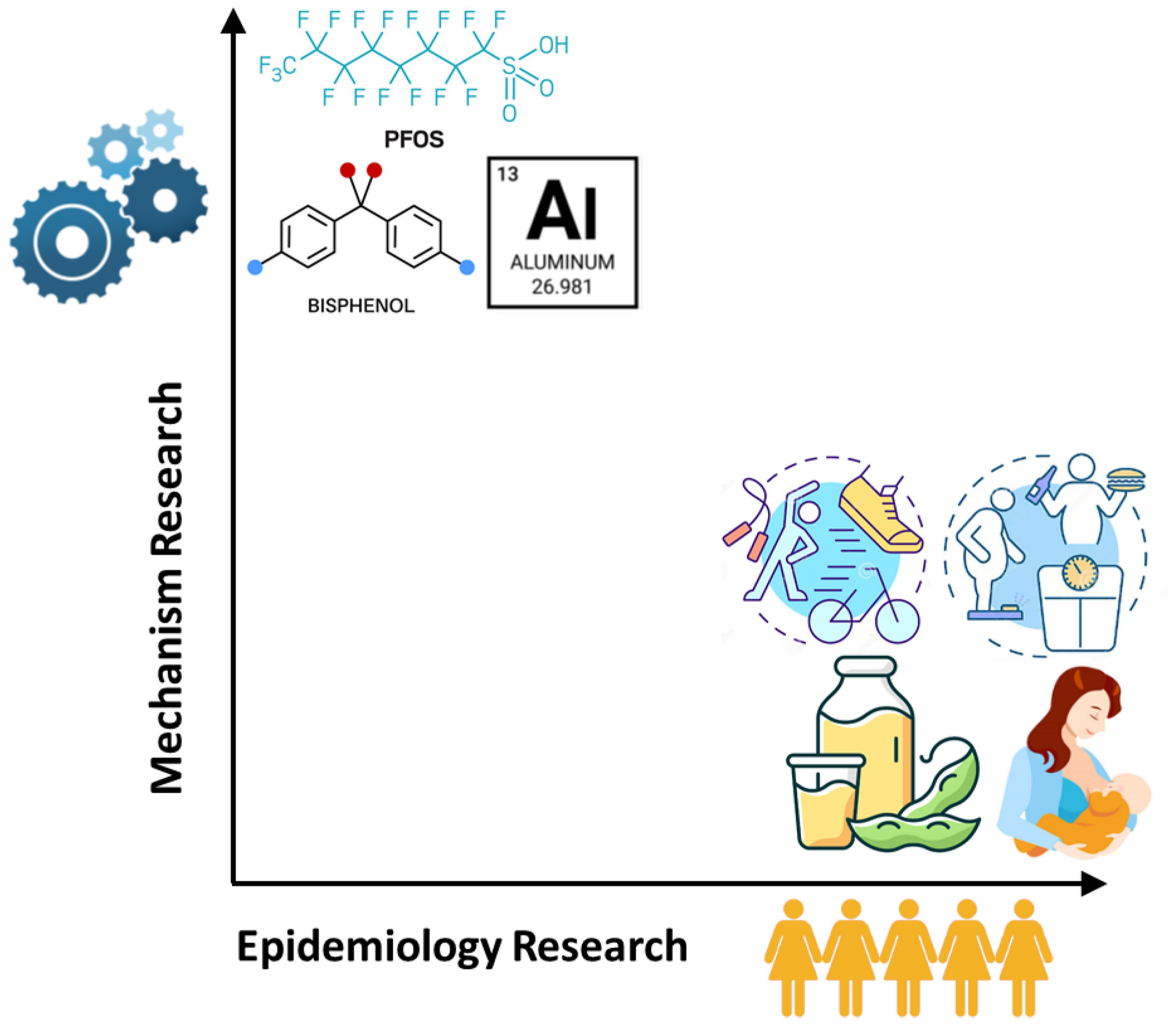
Breast Cancer Risk Factors Mechanism and Epidemiology Research. Chemicals such as bisphenols, PFOS and aluminium have extensive research into their mechanism of action in cell lines and animal models, but epidemiological studies have reported conflicting results. Health behaviours such as physical activity, weight control, consumption of soya in Asian populations and breastfeeding have consistent associations with breast cancer risk reduction but the underlying mechanisms of risk reduction need further research.

Knowledge of health behaviour‐related breast cancer risk factors is typically based on large, often prospective epidemiological studies providing consistent evidence on the level of risk reduction for those who breastfeed their children^6^ engage in physical activity^4^, ^5^ and control their weight.^7^ However, the mechanisms of risk reduction for these protective factors needs further research. In contrast, in the area of environmental chemical exposure there are many in vitro studies into the mechanism of action of individual chemicals and chemical mixtures in cancer cell models^8^, ^9^, ^10^, ^11^, ^12^ but large prospective epidemiological studies are lacking and often difficult to conduct^13^ (Figure 1). In part, this is because of the length of time that elapses between pollutant exposures and the eventual disease.

This review highlights the current state of play of breast cancer prevention research and recommends the types of breast cancer prevention studies that funding agencies need to prioritise.

## 2 WHICH ENVIRONMENTAL POLLUTANTS ARE LINKED TO BREAST CANCER RISK?

### 2.1 Endocrine disrupting chemicals

Breast cancer is strongly associated with lifetime exposure to endogenous estrogens.^14^ Humans are exposed to a multitude of chemicals in our environment.^15^ The exposome can be defined as the cumulative lifetime environmental exposure and related biological responses of an individual.^16^ Of particular concern for breast cancer risk are those chemicals that interact with the hormone system, or endocrine disrupting chemicals (EDCs). EDCs act as oestrogen receptor agonists,^17^ and induce effects that relate to breast cancer development.

#### 2.1.1 Bisphenols

Bisphenol A (BPA), an EDC, is found in polycarbonate‐containing plastics and resins.^18^ BPA leaches into food and drink from packaging (e.g. plastic bottles and plastic lining of cans) meaning most people are ingesting this EDC on a daily basis.^19^ BPA and other bisphenols have also been detected in feminine hygiene products, and as such there is potential dermal exposure.^20^ Recently, the European Food Safety Authority (EFSA) recommended reducing the tolerable daily intake (TDI) of BPA by 20,000‐fold (4 μg/kg to 0.2 ng/kg body weight per day).^18^ However, this reduction was driven by evidence that the human immune system was most sensitive to BPA exposure, and not breast cancer risk.^18^ The EFSA concluded that BPA is unlikely to be a genotoxic hazard through a direct mechanism.^18^ In contrast, rodent studies demonstrate that prenatal exposure to BPA may increase the propensity to develop mammary cancer during adulthood.^21^ However, a causal link between BPA and breast cancer remains equivocal because epidemiological studies have reported conflicting results.^22^

Regulatory restrictions of BPA have led industry to market ‘BPA‐free’ products by replacing BPA with other members of the bisphenol family. However, some BPA replacements (BPAF, BPB and BPZ), which are already in use and detectable in humans^23^ are even more potent oestrogenic EDCs than BPA in breast cancer cells, making them a ‘regrettable substitution’.^8^ It is therefore imperative that biomonitoring studies are conducted to determine the body burden of different bisphenols in human subjects and evaluate their combined toxicity, including oestrogenic EDC activity within a breast cancer context.

#### 2.1.2 PFAS

PFAS (per‐ and polyfluorinated alkyl substances), another group of EDCs are frequently used in consumer products because of their unique chemical and physical properties, including oil and water repellence, temperature and chemical resistance.^15^ PFAS are often called forever chemicals as they are extremely persistent and accumulate in humans, animals and the environment.^15^ PFAS are regulated because of their link to thyroid disease, increased cholesterol levels, liver damage, kidney and testicular cancer but not due to the risk of breast cancer.^15^ In general, the PFAS which are subject to regulation have been substituted with other short‐chain and polymeric PFAS. Regrettably, several of these alternative PFAS are also persistent in the environment.^15^

Exposure to substances like perfluoroctane sulfonate (PFOS), perfluorooctanoic acid (PFOA) induces proliferation and transforms normal human breast epithelial cells to a malignant phenotype through specific mechanisms that include altered levels of cell‐cycle regulators and epigenetic modifications.^11^, ^12^ However, a causal link between PFAS and breast cancer remains unclear because epidemiological studies have reported conflicting results.^24^

### 2.2 Aluminium

Occupational exposure to aluminium smelters increases the risk of bladder cancer. Aluminium itself, however, is currently not classified as a carcinogen.^25^ Aluminium is widely used in cosmetics as the active antiperspirant agent as well as in sunscreens. Epidemiological studies investigating potential associations between non‐occupational aluminium exposure and breast cancer incidence are scarce and have provided conflicting results.^26^ Aluminium has been shown to accumulate in breast tissue compared to blood or human milk, 6 μg/L in blood, 25 μg/L in human breastmilk and at 150 μg/L in Type I breast cysts.^27^ Aluminium also been shown to be at higher levels in nipple aspirate fluid of women with cancer (268 μg/L) compared to healthy controls (131 μg/L).^27^ This, combined with data showing increased incidence of breast cancer in the upper‐outer quadrant of the breast has led researchers to hypothesise that exposure to aluminium in antiperspirants is contributing to breast cancer risk.^27^

At concentrations close to those measured in the human breast (starting at 10 μM AlCl_3_x6H_2_O that is, 270 μg/L aluminium) this metal enters several mammalian cell types within 3 h and induces DNA double‐strand breaks (DSB) within 24 h of exposure, with very low concomitant cytotoxicity.^26^, ^28^ If aluminium is inducing genomic instability, it is also inducing one of the enabling hallmarks of cancer^29^ which drives carcinogenesis. Aluminium‐induced DSB could be particularly relevant to breast carcinogenesis, due to the known contribution of germline mutations in DSB repair genes to familial breast cancer and to the reported genetic/epigenetic inactivation of the same genes in a fraction of sporadic breast cancer.^22^

Research needed to understand aluminium's carcinogenic potential includes further assessment of its genotoxic effect in vivo. Further, prospective epidemiological studies comparing breast cancer incidence in aluminium‐exposed versus non‐exposed populations are also needed. However, the data accumulated so far are sufficient to consider restricting the use of aluminium, based on the precautionary principle.

### 2.3 Epidemiology and chemical mixtures

Epidemiological studies examining whether individual chemicals are implicated in breast cancer risk have in general produced inconclusive results such in the case of BPA, PFAS and aluminium.^22^, ^24^, ^26^ These observations often lead to the assumption that the concerns associated with the role of EDCs in breast cancer are unfounded, as their levels in tissues are not high enough to increase breast cancer risk. The problem with this assumption is that humans are concurrently exposed to large numbers of chemicals, most at low levels which might produce significant combination effects. Hence, the examination of one xenoestrogen at a time is likely to underestimate the combined risk from simultaneous exposures and fail to highlight a link between exposures and effects. However, laboratory studies have shown that multiple xenoestrogens, including mixtures of Bisphenols and PFAS, can work together to exhibit effects, even when combined at levels that alone are ineffective.^30^

The first case–control study to report an association between the total estrogenic load in adipose tissue and breast cancer used the TEXB‐α assay (organohalogenated xenoestrogens). Increased breast cancer risk associated with high levels of TEXB‐α was not found in the study population as a whole.^31^ However, high TEXB‐α was associated with increased breast cancer risk in postmenopausal women with body‐mass index (BMI) below the median (28.6 kg/m^2^) (OR: 5.67; 95% CI 1.59–20.21).^31^ A more recent study showed a strong positive association between serum xenoestrogenic load and breast cancer using the TEXB‐α and TEXB‐β (endogenous hormones and more polar xenoestrogens) assays.^32^ There was increased breast cancer risk with higher TEXB‐α (OR 3.45, CI 1.50–7.97) and TEXB‐β (OR 4.01, CI 1.88–8.56), comparing the lowest and highest tertiles of the dataset.^32^ While these studies provide evidence of a link between chemical exposures and breast cancer and highlight that this is an issue of exposure to multiple chemicals simultaneously, they cannot pinpoint specific chemicals that contribute to risks or the source of the environmental exposure. The TEXB‐α/β studies discussed here both collected their serum or breast/adipose prior to chemotherapy.^31^, ^32^ This is important as TEXB‐α/β levels in have been reported to increase in breast cancer patients after chemotherapy.^33^ Limitations of the TEXB‐α/β work include their retrospective study design.^31^, ^32^ Future validation should ideally be performed in prospective studies where samples are taken before diagnosis to establish a causative link.

Unfortunately, traditional epidemiology, with its focus on single chemicals or groups of similar chemicals, cannot fully capture the extent of breast cancer risk from chemical exposure. In a systematic review of polychlorinated biphenyls and breast cancer risk, four studies detected an association, four were null studies, and one even established breast cancer‐preventing effects.^34^ A similar picture emerged with other organochlorines, brominated and fluorinated organics.

These findings highlight the importance of a breast cancer risk epidemiology approach that considers the combined effects of the large variety and number of chemicals to which we are exposed, rather than single chemicals or groups of similar chemicals.

### 2.4 Early life exposures to EDCs

The developmental origins of health and disease (DOHaD) is a well‐established paradigm which recognises that predisposition to certain non‐communicable adult diseases is associated with disruptions to the foetal environment and foetal development.^35^ For example, maternal factors such as overnutrition, nutritional restriction, hypoxia and stress, are well known to be associated with obesity, type II diabetes and cardiovascular disease in adulthood.^36^ The concept that maternal exposure to certain chemicals and drugs during pregnancy can adversely affect foetal development is not new. The impacts of cigarette smoking and alcohol consumption during pregnancy are well known to have long‐term adverse health outcomes on prenatally exposed offspring.^37^, ^38^

Exposure to estrogenic chemicals during vulnerable stages of life can increase the risk of breast cancer. Some of the earliest evidence came from human populations exposed during gestation to the estrogenic pharmaceutical diethylstilbestrol (DES).^39^ For breast cancer occurring ≥40 years, there was increased risk associated with DES exposure (IRR 1.91, CI 1.09–3.33).^39^ The lessons learned from these so‐called DES daughters have now been extended to other estrogenic pollutants such as the pesticide dichlorodiphenyltrichloroethane (DDT). Human cohort studies have shown that gestational exposures to DDT significantly increase the risk of breast cancer (OR 3.7, CI 1.5–9.0),^40^ as well as other risk factors for breast cancer such as increased breast density.^41^

One major challenge that comes from these kinds of studies is the need for cohorts that evaluate environmental pollutant exposures during sensitive life stages, which often precede the age at which breast cancers develop by decades. For this reason, most of the human studies examining exogenous EDC exposure during gestation, like DES^39^ and DTT,^40^ have utilised human populations exposed accidentally to high concentrations of a specific chemical.

Due to the challenges of studying early life exposures to environmental pollutants in humans, they are often evaluated in controlled exposures to laboratory animals. Low, environmentally relevant exposures to EDCs (BPA, BPS and BPAF) in rodents during early development can increase the risk of mammary cancers in adulthood.^21^ Evidence suggests that EDCs can alter other aspects of breast health that are considered risk factors for breast cancer including an earlier timing of puberty and alterations to lactation.^17^ The evidence indicating that reproductive and breast health is negatively impacted by EDCs provides an important opportunity to protect public health via exposure mitigation.

### 2.5 Future environmental pollutant‐related breast cancer risk research priorities include

1. Prospective studies where samples are taken before a breast cancer diagnosis to establish a causative link.
2. Modern epidemiological approaches examining chemical mixtures of the kind pioneered by Ibarluzea et al.^31^ and Pastor‐Barriuso et al.^32^ using TEXB‐α/β combined with cutting‐edge analytical approaches to detect the chemicals that determine the internal load of xenoestrogens and other xenobiotics.
3. Assessment of real‐life exposure to environmental chemical mixtures during pregnancy, and their potential involvement in programming adverse foetal health outcomes in later life.

## 3 WHICH HEALTH‐BEHAVIOUR RELATED FACTORS ARE LINKED TO BREAST CANCER RISK?

In the UK, higher rates of breast cancer over the last 40 years have occurred alongside increases in potentially modifiable breast cancer risk factors. The 2021 NHS Health Survey for England found that 59% of women were either overweight or obese.^42^ This figure rises to 65% of women between the ages of 45 and 54, the age at which breast cancer incidence rises.^42^ In addition, 15% of women in England drink more than 14 units of alcohol per week, with highest levels of consumption in the 55–64 age group.^42^ Observational studies have consistently found associations with obesity and alcohol and the risk of breast cancer.^2^, ^43^, ^44^ Adherence to a Mediterranean diet, which typically includes legumes, cereals, fruits/nuts, vegetables, extra virgin olive oil and low amounts of red meat, poultry and dairy products, has been consistently linked to a reduction in the risk of cardiovascular disease^45^ but not breast cancer (RR 1.01, CI, 0.88–1.16).^46^

The Breast Cancer Prevention Conference featured talks relating to the latest evidence on soya, weight, breastfeeding and physical activity on breast cancer risk; as well as evidence on the impact of these factors on survival and quality of life (QoL) after a breast cancer diagnosis.

### 3.1 Soya

Isoflavones, naturally occurring compounds within soya, are similar in structure to human oestrogen and are classified as selective oestrogen receptor modulators (SERMs). Lower rates of breast cancer incidence in Asian countries, in comparison to Western populations, have been attributed to soya/isoflavone consumption. Meta‐analyses of epidemiological studies indicate that soya/isoflavone consumption is associated with a ~40% reduction in breast cancer risk in Asian women.^47^ This finding was consistent in both pre (OR 0.59, CI 0.48–0.69) and post‐menopausal women (OR 0.59, CI 0.44–0.74).^47^ Similar protective effects are not observed in Western populations,^47^ possibly due to the timing of exposure to soya foods. Soya is consumed in higher amounts and from a young age in Asian populations. A greater reduction in premenopausal breast cancer risk is observed in Asian women that consume a high‐soya diet during adolescence and maintain intake in adulthood.^48^

### 3.2 Weight

Most studies that assess breast cancer risk associated with body weight have assessed this based on BMI either at diagnosis or at start of entry to a cohort. However, the association is not straightforward with increasing BMI is associated with increased risk in post‐menopausal women (~35%), but with decreased risk in premenopausal women (~10%). The PROCAS (Predicting‐Risk‐Of‐Cancer‐At‐Screening) study recruited 47,042 women between 2009 and 2013. BMI was determined at baseline and (by recall) at age 20 years. With a median follow‐up of 5.6 years, 1142 breast cancers (post‐menopausal at entry: 829) occurred.^2^ Among post‐menopausal women at entry, BMI aged 20 years was inversely associated (HR per SD (3.23 kg) 0.87, CI 0.79–0.95), while absolute weight gain was associated with breast cancer (HR per SD (0.34 kg/year) 1.23, CI 1.14–1.32).^2^ For post‐menopausal women who had a recall BMI aged 20 years <23.4 kg/m^2^ (75th percentile), absolute weight gain was associated with breast cancer (HR per SD (12.2 kg) 1.31, CI = 1.21–1.42), but there were no associations for women with a recall BMI aged 20 years of >23.4 kg/m^2^ (*p* = 0.451). Adult weight gain increased post‐menopausal breast cancer risk only among women who were <23.4 kg/m^2^ aged 20 years, with those obese at age 20 being particularly protected.^2^ A further study showed that being obese at age 20 was associated with almost doubling of the risk premature deaths (HR 1.90, 95 CI 1.45–2.48). Although early adult obesity protects against breast cancer risk it is associated with much higher rates of early death.^3^

### 3.3 Breastfeeding

The relative risk of breast cancer is decreased by 4.3% (CI 2.9–5.8) for every 12 months of breastfeeding, which was in addition to the 7.0% (CI 5.0–9.0) decrease in risk observed for each birth.^6^ The decreased risk of breast cancer was the same in high and low income countries and did not vary with age, menopausal status, ethnic group or age at first birth.^6^ This data truly indicates that breastfeeding universally decreases breast‐cancer risk. In the context of a high‐income country such as the UK, a woman who has two children and breastfed for 12 months with each child will have reduced her risk of breast cancer by 8.6%.

Understanding which subtype of breast cancer has its risk reduced by breastfeeding will give insights into the mechanism of risk reduction. A meta‐analysis found no reduction in the risk for hormone‐receptor positive (HR+) breast cancer associated with breastfeeding, but found a 20% reduction in the risk of triple‐negative breast cancer (TNBC).^49^ The literature on the risk of HER2+ breast cancer and breastfeeding followed the data on pregnancy associated risk.^50^ Studies that found an increased risk of HER2+ breast cancer with pregnancy also reported a lower risk in women who breastfed.^50^ In contrast, the studies that found either no change or a decrease in risk of HER2+ breast cancer associated with pregnancy also found no change with breastfeeding.^50^

Consistent with the reduction in risk in TNBC with breastfeeding; women with BRCA1 mutations who breastfed for more than 1 year were found to have a 22%–50% reduced risk of breast cancer than those who never breastfed.^51^, ^52^ In contrast, no decreased risk associated with breastfeeding for women with BRCA2 mutations has been established in several studies.^52^, ^53^ This initially seems counter‐intuitive given the similarity in function between the BRCA1 and BRCA2 proteins. However, the loss of BRCA1 or BRCA2 has different effects on breast tumour pathology^54^ as well as tumour cell reliance on oestrogen receptor signalling and tumour microenvironment.^55^ Consequently, it has been suggested that they now should be regarded as different tumour types with different treatments.^55^

Elevated hormone levels during pregnancy causes the ductal system of the breast to expand and the alveolar epithelium which produces milk increases in size.^56^ When breastfeeding stops there is a regression in the breast tissue but there is no substantial reduction of the mammary glands.^57^ The lobules in the breast involute as a woman ages with a reduction in the number of alveoli. Over time there is a replacement of the mammary glands with fatty tissue.^57^

One theory on how breastfeeding reduces the risk of breast cancer is by detoxifying the breast of EDCs or other chemicals that have built up in breast tissue over a woman's lifetime.^58^ However, the lack of a clear reduction in risk of HR+ positive breast cancer with breastfeeding suggests that breastfeeding is not reducing the amounts of EDCs in breast tissue. In contrast, the reduction in risk in TNBC suggests that detoxification of other genotoxic chemicals could play a role but further research is needed.

A systematic review has shown an inverse association has also been found between maternal PFAS exposure and breastfeeding duration.^59^ The association is present after adjusting for confounding factors that affect breastfeeding such as prior history of breastfeeding, parity, foetal age, maternal age, BMI and education. These studies suggest that PFAS is affecting women's ability to lactate and perceived insufficient milk supply is one of the top reasons that women stop breastfeeding. However, true‐milk insufficiency effects only around 10% of mothers.^50^

### 3.4 Physical activity

A meta‐analysis of 10 prospective cohort studies including 816,668 females shows the risk of developing breast cancer is 10% lower (hazard ratio, HR: 0.90, 95% confidence intervals, CI: 0.87–0.93) in the 90th compared to the 10th percentile of self‐reported leisure‐time physical activity.^4^ The magnitude of this association was largely unchanged and remained statistically significant when adjusting for BMI and other risk factors (e.g. including parity, age at menarche and menopause).^4^ Of interest, the study also performed an analysis of breast cancer risk by breast cancer subtype. The risk of oestrogen receptor positive (ER+) breast cancer was largely the same for the whole cohort as this is the most common subtype (HR 0.89, CI, 0.82–0.97).^4^ There was a greater reduction in risk for ER‐ cancers (HR 0.72, CI, 0.59–0.88), which would include PR+, HER2+ and TNBC.^4^

A dose–response meta‐analysis of eight prospective cohort studies including 403,932 females, with similar adjustment for potentially confounding variables, shows that an almost identical reduction in risk of breast cancer (HR 0.90, CI 0.86–0.94) is brought about by undertaking 5 h of moderate‐intensity physical activity each week, and risk decreases further (HR 0.86, CI 0.82–0.90) if activity levels are doubled.^5^ Causal evidence from randomised and controlled trials (RCTs) examining whether physical activity and exercise throughout life reduces breast cancer risk is not available and remains an unmet and important challenge.^60^ However, assuming an invasive breast cancer incidence of 1.7% over 5 years, it has been estimated that 20,000–45,000 females would need to be randomised to a 5‐year exercise intervention RCT to detect the reduction in risk shown by epidemiological studies.^60^ Another important challenge is determining biological mechanisms underlying reduced breast cancer risk with a physically‐active lifestyle. Compared to the scale of RCTs needed to detect disease incidence, RCTs recruiting females considered to be at high risk of breast cancer, have been designed with a more manageable number of participants (*n* = 139).^61^ However, these trials—examining whether exercise training influences plausible mechanisms by measuring changes in markers of risk (e.g. sex hormones, inflammatory proteins, adipokines and body composition)—have generally reported no changes. Indeed, as reviewed recently^62^ the most likely mechanisms by which exercise influences breast cancer risk (i.e. detection and elimination of pre‐cancer cells by T cells) have not been thoroughly investigated.

### 3.5 Health behaviour‐related risk factors after a breast cancer diagnosis

Women with a breast cancer diagnosis often seek lifestyle information for improving their QoL, prevention of recurrence and survival. In breast cancer survivors, there was strong probable evidence that physical activity improves QoL.^63^ There was strong probable evidence that a high post‐diagnosis BMI increases the risk of all‐cause mortality, breast cancer‐specific mortality and a second breast cancer diagnosis.^64^ The respective risk ratios (RR) and CIs per 5 kg/m^2^ BMI were 1.07 (1.05–1.10), 1.10 (1.06–1.14) and 1.14 (1.04–1.26).^64^ High post‐diagnosis dietary fibre intake may lower all‐cause mortality (RR per 10 g/day: 0.87, CI 0.80–0.94).^65^

Animal studies in which the soya isoflavone genistein stimulated oestrogen‐sensitive breast cancer cell growth have raised concern over the safety of soya consumption for breast cancer patients. However, rodents metabolise isoflavones differently than humans,^66^ and these findings are not supported by human studies. Epidemiological studies in breast‐cancer survivors demonstrate that soya does not adversely affect recurrence, or mortality^65^; respective RR per 2 mg/day: 0.75 (0.61–0.92) and 0.83 (0.64–1.07). Furthermore, soya/isoflavone consumption does not appear to interfere with tamoxifen therapy.^67^ Current evidence supports the safety of moderate soya consumption for breast cancer survivors and women at high risk of breast cancer. Randomised clinical trials investigating the effect of soya/isoflavones on breast cancer recurrence and mortality are needed to confirm if soya/isoflavone consumption improves prognosis.

There are methodological limitations in cancer survival studies, yet the findings support the development of health behaviour guidelines for breast cancer survivors to avoid weight gain, be physically active and eat a healthy diet, within the limits of their ability and specific medical advice.

### 3.6 How can we promote risk reducing behaviours?

Achieving population‐wide healthy weight, physical activity and control of alcohol and smoking has the potential to reduce rates of breast cancer. Estimates of how much range from 15% to 30% in western populations.^43^, ^68^ Population level approaches to promote healthy behaviour include food industry regulation with advertising, labelling, reducing the cost of healthy food and sugar or fat taxes on unhealthy foods. There is also a role for scalable supportive programmes targeted to those at high risk, that is, women with a family history for whom weight and health behaviours can exert a greater effect on absolute risk than women at population risk. There is some evidence that women with overweight/obesity who are high‐risk (estimated lifetime risk of > or = 17%) are more likely to engage with a disease prevention weight‐loss programme compared to their counterparts who were identified at or below population risk.^69^ Breast cancer risk can start accumulating from childhood. There is an unmet need for strategies and programmes to initiate early prevention of breast cancer for the next generation.^70^

### 3.7 Future health behaviour‐related breast cancer risk research priorities include

1. Understanding the underlying mechanisms of breast cancer prevention in women who breastfeed their children, are physically active and control their weight may lead to novel chemo‐preventive or targeted breast‐cancer treatments.
2. Generating more robust data, in an ethnically‐diverse cohort, to indicate the most relevant health behaviours to lower risk. It is not feasible for observational associations to be verified in randomised breast cancer incidence trials due to sample size considerations.^60^ These data can be strengthened by assessing causality with Mendelian randomisation studies.^71^
3. The interactions between genetic and health‐behaviour based breast cancer risk factors for mutation carriers/those with high polygenic breast cancer risk scores.
4. Identifying easily measured surrogate markers of breast cancer risk which are modified by health behaviours, that is, weight loss and increased physical activity.
5. There is a need to implement what we know. This could test health behaviour change programmes in individuals at risk including those attending breast screening, high‐risk clinics and work with families at risk to initiate early prevention. Evaluation would include potential benefits and harms of such programmes including any effects on screening attendance or effects on health inequalities.

## 4 CONCLUSIONS

A broader perspective on breast cancer risk and prevention research is required including both health behaviour‐related risk and environmental exposure to chemicals. Behaviour‐related research needs to focus on implementation of what we already know as well as deeper understanding of the mechanisms of breast cancer prevention. Research into chemical exposure needs to focus on chemical mixtures and larger prospective epidemiological studies in order to test the effects on breast cancer risk. Research funding available in this field is typically not of an amount large enough to conduct long‐term prospective studies. On such a much better‐founded scientific basis, more effective prevention strategies that integrate insights from health behaviour‐related risks and pollution research could be developed.

## AUTHOR CONTRIBUTIONS

**Britta Stordal:** Conceptualization (lead); project administration (lead); writing – original draft (lead); writing – review and editing (lead). **Michelle Harvie:** Writing – original draft (supporting); writing – review and editing (supporting). **Michael N. Antoniou:** Writing – original draft (supporting); writing – review and editing (supporting). **Michelle Bellingham:** Writing – original draft (supporting); writing – review and editing (supporting). **Doris S. M. Chan:** Writing – original draft (supporting); writing – review and editing (supporting). **Philippa Darbre:** Writing – original draft (supporting); writing – review and editing (supporting). **Oskar Karlsson:** Writing – original draft (supporting); writing – review and editing (supporting). **Andreas Kortenkamp:** Writing – original draft (supporting); writing – review and editing (supporting). **Pamela Magee:** Writing – original draft (supporting); writing – review and editing (supporting). **Stefano Mandriota:** Writing – original draft (supporting); writing – review and editing (supporting). **Elisabete Silva:** Writing – original draft (supporting); writing – review and editing (supporting). **James E. Turner:** Writing – original draft (supporting); writing – review and editing (supporting). **Laura N. Vandenberg:** Writing – original draft (supporting); writing – review and editing (supporting). **D. Gareth Evans:** Writing – original draft (supporting); writing – review and editing (supporting).

## FUNDING INFORMATION

Breast Cancer UK as the funder of the Breast Cancer Prevention Conference at Middlesex University in June 2023. D Gareth Evans is supported by the NIHR Manchester Biomedical Research Centre (IS‐BRC‐1215‐20007) and Cancer Research UK ACED Alliance Early Detection Centre C19941/A27859.

## CONFLICT OF INTEREST STATEMENT

The authors declare that they have no competing interests.

## SCOPE AND ORGANISING COMMITTEE

The Breast Cancer UK – Breast Cancer Prevention Conference scope was to cover developments in environmental chemical exposure and health behaviour related breast cancer risk. Chemoprevention and surgical risk reduction was outside the scope of the conference. The organising committee was authors B.S., E.S, J.T, P.D., as well as Prof. Valarie Speirs (University of Aberdeen), Ms. Thalie Martini and Ms. Kerri Palmer (Breast Cancer UK). All authors on the manuscript gave oral presentations at the conference. Keynote presentations were given by L.V. and M.H.

## Data Availability

Not applicable.
